# A nationwide registry-based cohort study of the association between childhood dental caries and gingivitis with type 2 diabetes in adulthood

**DOI:** 10.1007/s00592-024-02437-4

**Published:** 2025-01-13

**Authors:** Nikoline Nygaard, Anne Kirstine Eriksen, Lars Ängquist, Daniel Belstrøm, Evelina Stankevic, Torben Hansen, Anja Olsen, Merete Markvart

**Affiliations:** 1 Section for Clinical Oral Microbiology, Department of Odontology, Faculty of Health and Medical Sciences, University of Copenhagen, Nørre Allé 20, 2200 Copenhagen, Denmark; 2 Danish Cancer Institute, Diet, Cancer and Health, Strandboulevarden 49, 2100 Copenhagen, Denmark; 3 Novo Nordisk Foundation Center for Basic Metabolic Research, Faculty of Health and Medical Sciences, University of Copenhagen, Blegdamsvej 3B, 2200 Copenhagen, Denmark

**Keywords:** Cohort studies, Dental caries, Diabetes mellitus, Type 2, Gingivitis

## Abstract

**Background:**

Evidence suggests a bidirectional relationship between oral health status and type 2 diabetes (T2D) in adults. Studies on associations between childhood oral health and T2D in adulthood are lacking.

**Methods:**

This is a nationwide Danish registry-based cohort study of individuals born between 1963 and 1972, having at least one registration in the National Child Odontology Registry between 1972 and 1987 (n = 627,758). Follow-up lasted from 1995 to 2018. Main exposure variables were the highest achieved levels of dental caries and gingivitis between 1972 and 1987. The outcome was T2D diagnosis during follow-up. Data was analyzed using Cox-regression, stratified on sex, with age as the underlying timescale and highest achieved level of education between age 25–30 years as Cox-strata. Main analyses were conducted with and without age-restrictions (T2D diagnosis before/after age 40).

**Results:**

Compared to lowest-level references, high levels of gingivitis associated with increased hazard ratios (HRs) of T2D in both males (HR [95% confidence interval]: 1.59 [1.47; 1.72]) and females (1.87 [1.68; 2.08]), as did severe dental caries (males: (1.15 [1.04; 1.27], in females: 1.19 [1.06; 1.35]). Below age 40, gingivitis associated with increased HRs in males (1.84 ([1.58; 2.15]) and females (1.94 [1.63; 2.30]). Above age 40, both exposures displayed higher HRs in males (high gingivitis: 1.52 [1.39; 1.66] vs. severe caries: 1.23 [1.09; 1.38]) and females (1.83 [1.59; 2.10] vs. 1.37 [1.17; 1.59]).

**Conclusions:**

Data suggest an association between childhood dental caries and gingivitis with risk of receiving a T2D diagnosis in adulthood. However, results are affected by residual confounding warranting further studies.

**Supplementary Information:**

The online version contains supplementary material available at 10.1007/s00592-024-02437-4.

## Introduction

Evidence points to a bidirectional relationship between oral health status and type 2 diabetes (T2D) in adults. Dental caries (DC), periodontitis, and gingivitis are the most common oral diseases, as well as amongst the most prevalent diseases worldwide [1]. DC is as a localized, chemical dissolution of a tooth surface caused by the metabolic activity of the tooth surface microbiota [2]. Periodontitis is an aggressive inflammation of the tooth-supporting tissues, which can eventually lead to tooth loss [3]. Gingivitis covers a wide range of disease states ranging from light bleeding by a single tooth to whole-mouth bleeding and inflammation, and can be seen as a precursor of periodontitis [4–6].

The association between periodontitis and T2D is the most well documented, suggesting that periodontitis treatment improves glycemic control in individuals with T2D [7, 8]. The association between gingivitis and T2D has not been as extensively explored. Nevertheless, studies of 2.144–93.647 individuals find higher levels of gingivitis in individuals with T2D compared to controls [9, 10]. Both gingivitis and periodontitis are inflammation-mediated diseases [5], and correspondingly systemic low-grade inflammation has been suggested as the link to systemic diseases like obesity and T2D [11]. Evidence for an association between DC and T2D is limited, but some studies indicate that individuals with poorly controlled T2D have higher rates of DC when compared to metabolically healthy individuals [12]. Evidence on whether DC and gingivitis can predispose to the development of diabetes is lacking, and the mechanisms potentially linking DC with systemic disease remain to be uncovered.

DC and gingivitis are highly prevalent in children and adolescents [1, 13]. Given the associations observed in adults, DC and gingivitis in childhood potentially have a lifelong impact on systemic health. A study of the association between childhood oral health and the adulthood risk of cardiovascular disease in 755 individuals found a higher prevalence of cardiovascular risk factors, risk of subclinical atherosclerosis and metabolic syndrome in adulthood, amongst those with oral infections or inflammation in childhood [14, 15]. However, there is a lack of studies on the association between childhood oral health and T2D in adulthood.

Data from The Danish National Child Odontology Register (SCOR) combined with data from the Register of Selected Chronic Diseases (RUKS) and the Civil Registration System (CRS) presents a unique opportunity to investigate the link between childhood oral disease and T2D in adulthood. In this paper we utilize this to first test the hypothesis that there is an association between childhood experience of DC and gingivitis with adulthood T2D. Secondarily, we hypothesize that the age at which the highest registered level of disease occurs, as well as the duration and severity of oral disease is associated with the incidence of T2D, which we examine in a subset of the study population.

## Methods

This is a nationwide Danish registry-based cohort study of individuals born between 1963 and 1972, and with at least one registration in SCOR between 1972 and 1987. Individuals were followed through registry data from January 1st, 1995, or age 25 (whichever occurred last), and were registered with an event upon receiving a diagnosis of T2D or censored upon first instance of either a diagnosis with type 1 diabetes (T1D), death, disappearance, emigration, or by the end of follow-up on December 31st, 2018.

### Registries

**Fig. 1:**
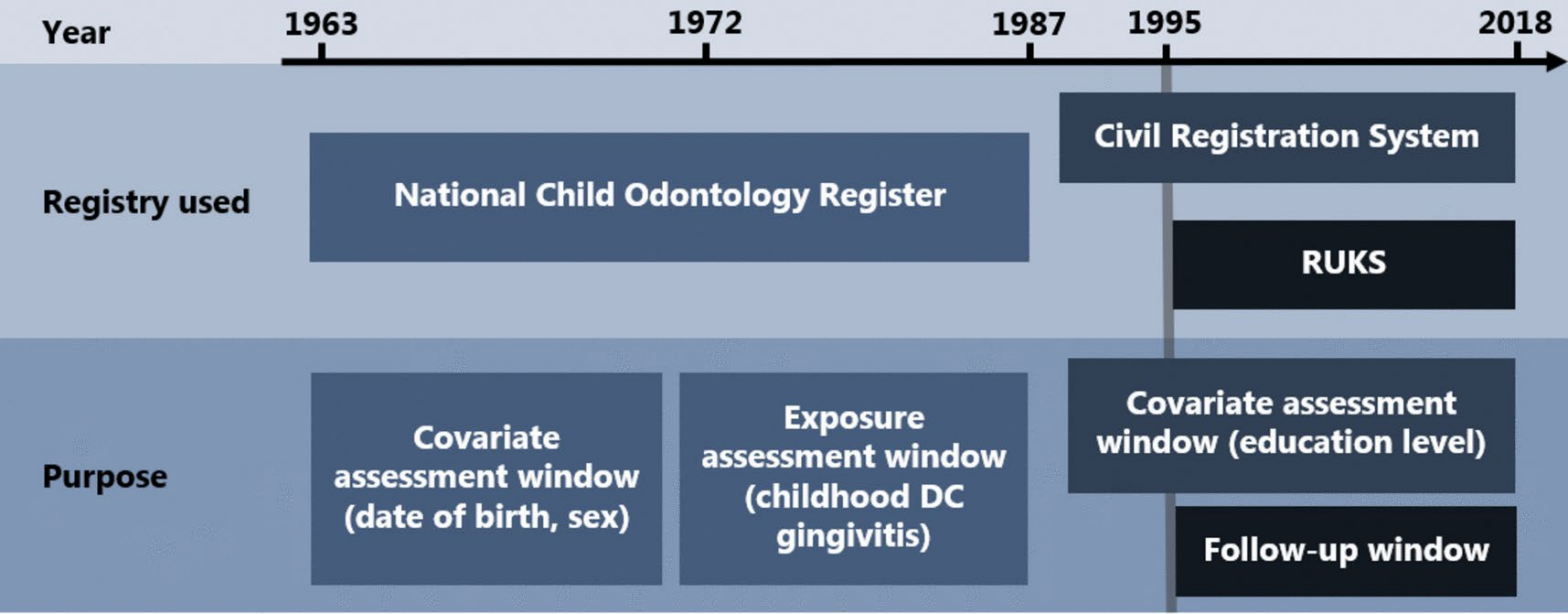
Data extraction process. *RUKS* Register of Selected Chronic Diseases

Information was gathered through linkage of relevant registries using the unique Danish personal identification number (CPR-number): (1) SCOR data on childhood DC and gingivitis from 1972 to 1987, date of birth, and sex. (2) RUKS for data on diagnosis of T1D and T2D between January 1st, 1995, and December 31st, 2018. To be registered with T1D or T2D in RUKS either two relevant diagnoses (ICD-8/ICD-10: 249/E10 [T1D], 250/E11 [T2D]), or at least two registered purchases of antidiabetic prescription drugs are needed. A detailed description of the criteria for being registered with diabetes in RUKS can be found elsewhere [16]. For T1D, we identified diagnosed individuals to be able to censor them. 3) CRS provided data on status (alive, dead, disappeared, or emigrated from August 8th, 1967, to December 31st, 2018, and educational status from December 1st, 1981, to September 1st, 2016 (Fig. 1).

### Exclusion criteria

**Fig. 2:**
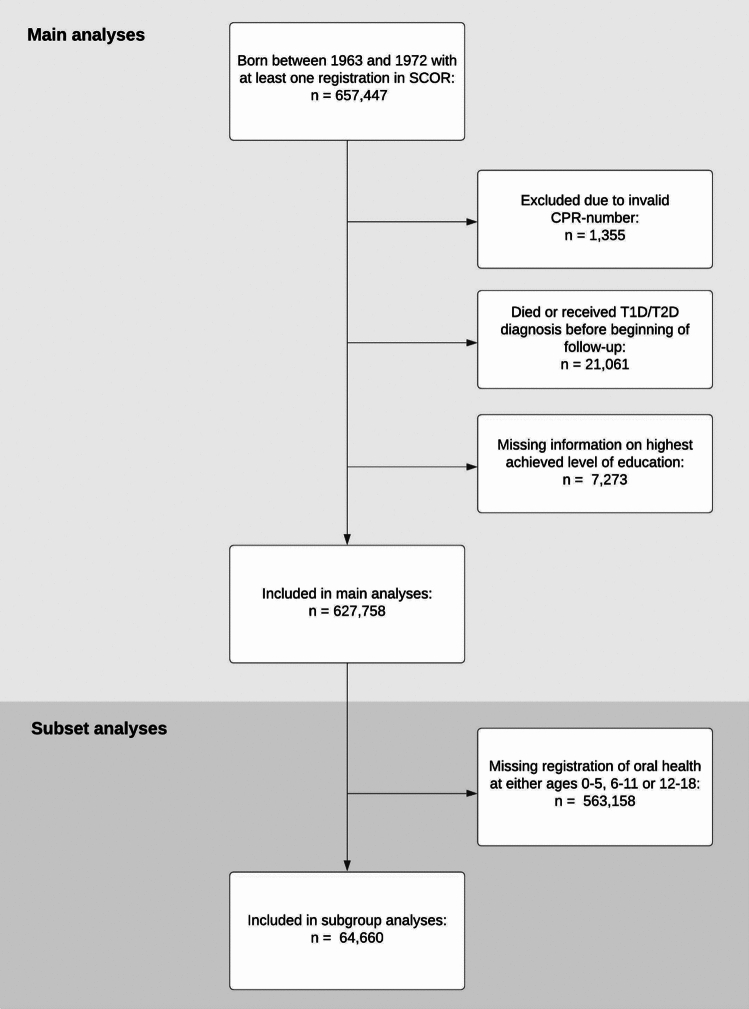
Selection of study participants. Flowchart of the selection of study participants for main and secondary (subset) analyses

Individuals were excluded from the study if they had been registered with codes denoting a faulty or invalid CPR-number, or if they had an event before beginning of their follow-up. Individuals without a registration of their level of education between ages 25 and 30 were also excluded from the study. For secondary analyses a subset of the study population with at least one registration at both ages 0–5, 6–11 and 12–18 were used (Fig. 2).

### Variables used in main analyses

Educational length was grouped into:

- Short: Mandatory schooling between ages 6–16.
- Medium: Secondary school and vocational education.
- Long: Higher education of any duration.

Based on this classification we used the highest attained level of education observed during ages 25–30, which should be close to the individuals’ final adult education level.

**Fig. 3:**
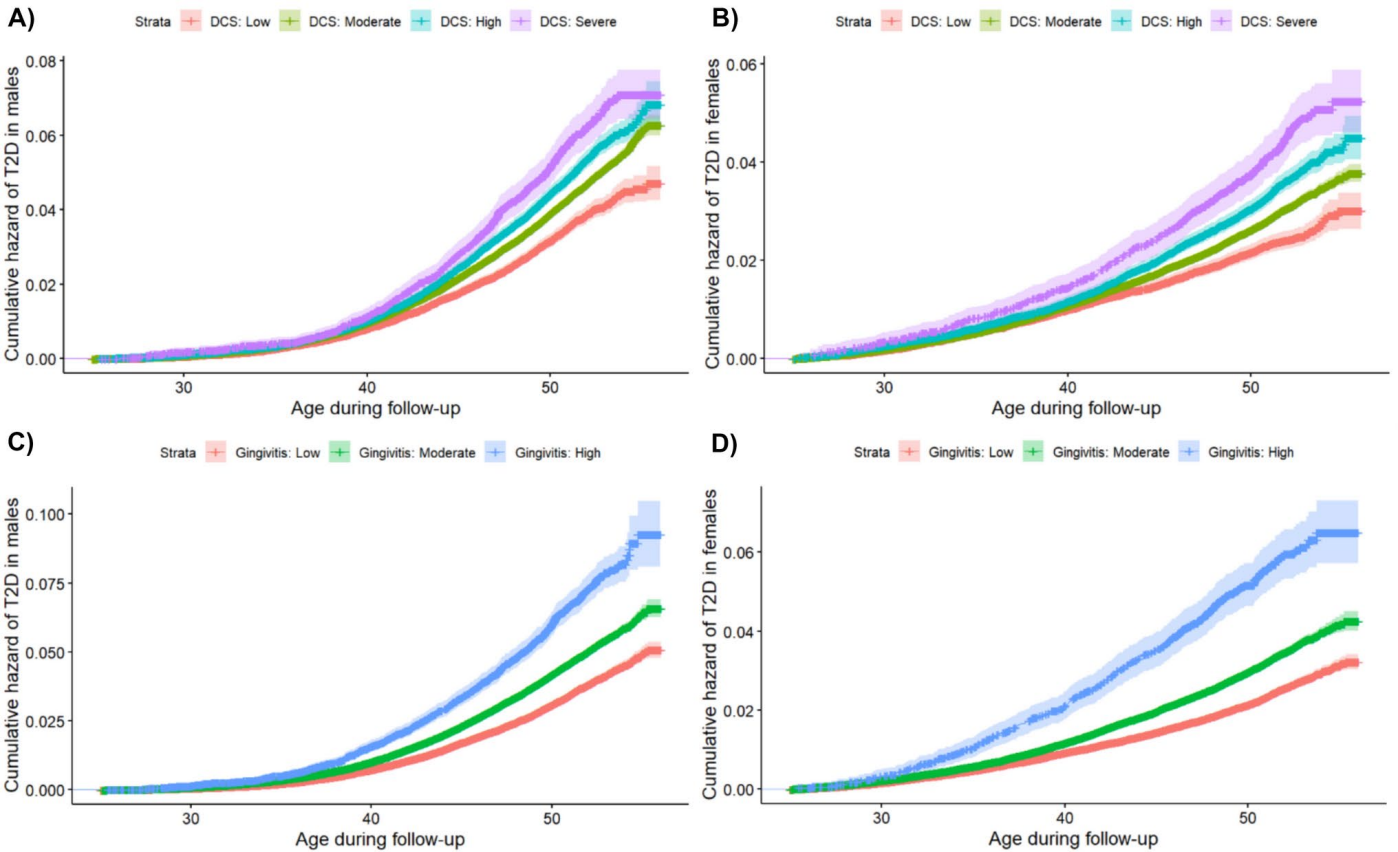
Plots of the cumulative hazard of T2D based on childhood DCS and GS, stratified by sex. Panel **A** The cumulative hazard of T2D in males based on the highest registered childhood DCS. Panel **B** The cumulative hazard of T2D in females based on the highest registered childhood DCS. Panel **C** The cumulative hazard of T2D in males based on the highest registered childhood GS. Panel **D** The cumulative hazard of T2D in females based on the highest registered childhood GS. *DCS* dental caries score, *GS* gingivitis score

Gingivitis was recorded using the Silness and Löe gingival index [4], and measured on four teeth (52, 55, 72 and 75 in the temporary dentition, 12, 16, 32 and 36 in the permanent dentition). An index-score ranging from 0 to 12 for the severity of gingivitis at each visit to the dentist was obtained by summing the index-score for each of the examined teeth [17]. The index-scores were 0 (healthy gingiva), 1 (slight changes in color and structure of gingiva but no bleeding), 2 (moderately inflamed gingiva, bleeding on probing), 3 (severely inflamed gingiva, bleeding, and ulceration). Based on odontological considerations this measure was categorized into gingivitis score (GS), using the highest registered sum index-score at any visit:

- Low: Score of 0–4.
- Moderate: Score of 5–8.
- High: Score of 9–12.

The scoring of DC in SCOR has been described in detail previously [17]. The measure of DC used in this study is the number of destroyed, missing, and filled teeth in either the primary, permanent, or mixed dentition (dmft/DMFT). This was calculated for each visit as the sum of codes denoting cavity, filling, and extraction due to DC. Based on odontological considerations the dmft/DMFT measures were used as a categorical variable, dental caries score (DCS), using the highest registered dmft/DMFT in any dentition at any point in time:

- Low: 0–4 affected teeth.
- Moderate: 5–12 affected teeth.
- High: 13–16 affected teeth.
- Severe: ≥ 17 affected teeth.

### Variables used in secondary analyses

For the secondary analyses we calculated the duration of DC and gingivitis by summing the number of periods in time (between ages 0–5, 6–11 and 12–18, roughly corresponding to the temporary, mixed, and permanent dentition), during which an individual had been registered as having DC (dmft/DMFT > 0) and gingivitis (gingivitis index-score > 0), resulting in a score from 0 to 3 for each disease. For the same subset of the study population, total disease severity over time was grouped based on the level of disease between the three examined timepoints, for gingivitis:

- Low: gingivitis index score ≤ 4 at all 3 age ranges.
- Moderate: gingivitis index score of 5–8 by at least one age range, no score ≥ 9.
- High: gingivitis index score ≥ 9 by at least one age range.

For DC:

- Low: dmft/DMFT ≤ 4 at all 3 age ranges.
- Moderate: dmft/DMFT of 5–12 by at least one age range, no dmft/DMFT ≥ 13.
- High: dmft/DMFT ≥ 13 by at least one age range.

### Statistical methods 

Baselines characteristics were described using frequencies and percentages for categorical variables and means and standard deviations for continuous variables.

Data analyses were done using Cox-regression modelling. Martingale residuals were plotted to assess the functional form and outliers for all continuous variables. To assess whether the Cox model assumptions were met Schoenfeld residuals, log–log curves, and cumulative hazards were plotted. The log–log curves for DCS and sex, respectively, were not parallel before age 40. Ideally this would have been solved by doing an interaction with time, however, due to limitations of the server we were working on, this was not possible. Instead, we examined whether there were any substantial time-dependent associations by running all analyses separately for males and females. Thus, primary analyses were run separately for time-at-risk below and above age 40 years. Further, the education-variable did not live up to the proportional-hazards assumption. The variable was used as strata in our models, allowing for differing baseline hazards between groups with differing education levels, thereby implicitly adjusting for the confounding effect of education in the models. Hazard ratios (HR) and 95% confidence intervals for the association between DCS and GS were produced using Cox-regression analysis with age as the underlying timevariable (see Fig. 3). 

All models were stratified by sex as we theorized that sex may moderate any associations between oral health and T2D due the protective effect of estrogen in adult females [18].

Main analyses:

Model (1) DCS + GS + strata (highest achieved level of education).

Model (2) DCS + GS + DCS * GS + strata (highest achieved level of education).

Secondary analyses:

Model (1) Age at highest registered DCS + duration of DC + severity over time of DC + strata(highest achieved level of education).

Model (2) Age at highest registered GS + duration of GS + severity over time of GS + strata(highest achieved level of education).

All analyses were conducted using the packages *prodlim* [19], *survival* [20], *timereg* [21] and *mets* [22] in R v.4.3.1 [23] with Rstudio (IDE version 2023.09.1 + 494). Reporting followed STROBE guidelines.

## Results

### Study size and participants

After removing individuals with missing information on educational level (n = 7,273), a total of 627,758 individuals were included in the study population (Fig. 1). The subset study population used for secondary analyses of the effect of oral health at different ages and disease severity over time, included 64,660 individuals with registrations within each age-range, 0–5, 6–11 and 12–18 years.

In total, 12,252 (3.8%) males and 7,990 (2.6%) females were diagnosed with incident T2D (Table 1). Most individuals regardless of sex and T2D diagnosis had a moderate DCS and GS as their highest registered level of disease (all groups: 46.1–67.7%). The percentage of individuals with a high to severe level of DCS was greater amongst individuals diagnosed with T2D (approximately 23% in those with T2D to 18.5% in the study population) but did not differ greatly between sexes. More females with T2D had a high GS (4.9%) compared to those with no T2D (2.5%). In males 7.2% of those with T2D had a high GS compared to 4.7% in the study population (for visualizations see supplementary Figs. 2–3). Amongst those diagnosed with T2D 36.8% had a low level of education, in the study population this was 21.2%.

**Table 1:** Descriptive characteristics of the study population stratified by sex (see next page)

	Males		Females	
	Study population	T2D	Study population	T2D
Number of individuals (%)	321,933 (100)	12,252 (3.8)	305,825 (100)	7,990 (2.6)
Follow-up age, mean (SD) years	49.4 (4.4)	43.8 (5.6)	49.7 (4.1)	41.7 (6.6)
Follow-up time, mean (SD) years	22.5 (3.8)	16.4 (5.2)	22.8 (3.3)	14.4 (6.1)
Education, N (%)				
Low	72,255 (22.4)	4,440 (36.2)	63,972 (20.9)	3,015 (37.5)
Medium	166,194 (51.6)	6,314 (51.5)	141,623 (46.3)	3,704(46.1)
High	83,484 (25.9)	1,498 (12.2)	100,230(32.8)	1,312 (16.3),
Any DC, N (%)	314,437 (97.7)	12,075 (98.4)	299,984 (98.1)	7,890 (98.8)
Highest DC, mean (SD)	9.13 (4.2)	9.72 (4.2)	9.27 (4.1)	9.95 (4.1)
DCS, N (%)				
Low	48,442 (15.1)	1,382 (11.3)	41,552 (13.6)	821 (10.3)
Moderate	214,039 (66.5)	8,128 (66.3)	207,179 (67.7)	5,306 (66.4)
High	48,993 (15.2)	2,182 (17.8)	46,677 (15.3)	1,449 (18.1)
Severe	10,459 (3.3)	560 (4.6)	10,417 (3.4)	414 (5.2)
Any gingivitis, N (%)	312,276 (97.0)	11,937 (97.4)	293,199 (98.9)	7,696 (96.3)
Highest gingivitis, mean (SD)	5.75 (2.3)	6.17 (2.3)	5.13 (2.2)	5.62 (2.3)
GS, N (%)				
Low	103,511 (32.2)	3,207 (26.2)	130,786 (42.8)	2,803 (35.1)
Medium	203,402 (63.2)	8,165 (66.6)	167,312 (54.7)	4,799 (60.1)
High	15,020 (4.7)	880 (7.2)	7,727 (2.5)	388 (4.9)
Number of individuals in subset analyses (%)	32,703 (100)	867 (2.7)	31,993 (100)	661 (2.1)
Age at highest recorded DCS, N (%)				
< 12 years	27,125 (83.0)	721 (83.2)	26,705 (83.5)	554 (82.3)
≥ 12 years	5,542 (16.9)	146 (16.8)	5,288 (16.5)	117 (17.7)
DC duration, N (%)				
0–2 periods	10,248 (31.4)	215 (24.8)	9,733 (30.4)	182 (27.5)
3 periods	22,419 (68.6)	652 (75.2)	22,260 (69.6)	479 (72.5)
DCS severity over time, N (%)				
Low	8,039 (24.6)	147 (16.9)	7,463 (23.3)	142 (21.5)
Moderate	17,374 (53.2)	483 (55.7)	17,647 (55.2)	355 (53.7)
High—Severe	7,254 (22.2)	237 (27.3)	6,883 (21.5)	164 (24.8)
Age at highest GS, N (%)				
< 12 years	25,087 (76.8)	630 (72.7)	26,837 (83.9)	517 (78.2)
≥ 12 years	7,580 (23.2)	237 (27.3)	5,156 (16.1)	144 (21.8)
Gingivitis duration, N (%)				
0–2 periods	10,079 (30.9)	213 (24.6)	11,120 (34.8)	208 (31.5)
3 periods	22,588 (69.2)	654 (75.3)	20,873 (65.2)	453 (68.5)
GS severity over time, N (%)				
Low	9,088 (27.8)	179 (20.7)	12,155 (37.9)	211 (31.9)
Moderate	22,147 (67.8)	625 (72.1)	19,050 (59.5)	425 (64.3)
High	1,432 (4.4)	63 (7.3)	788 (2.5)	25 (3.8)

*DC* dental caries, *DCS* dental caries score, *GS* gingivitis score, *N* number, *SD* standard deviations

In the subset study population 55.7%-75.3% had DC and gingivitis for the duration of their time in SCOR regardless of T2D diagnosis, but with more than 72% having their highest registered DCS and GS before age 12.

### The association between T2D, dental caries and gingivitis

Analysis with no age-restriction showed that having severe DCS compared to a low DCS in childhood was associated with the incidence of T2D in adulthood (HR = 1.19, CI: 1.06; 1.35 in females and HR = 1.15, CI:1.04; 1.27 in males) (Table 2, unadjusted estimates in Supplementary Table 1). Likewise, a high GS was associated with a higher incidence of T2D compared to a low GS (HR = 1.87, CI: 1.68; 2.08 in females and HR = 1.59, CI: 1.47; 1.72 in males). Amongst those diagnosed with T2D before age 40, the HR of T2D in both sexes was higher with increasing GS, while the incidence of T2D across levels of DCS were similar. Amongst those diagnosed after age 40 the incidence of T2D was higher in those with severe compared to low DCS (HR = 1.37, CI: 1.17; 1.59 in females and HR = 1.23, CI: 1.09; 1.38 in males), and a high compared low GS (HR = 1.83, CI: 1.59; 2.10 in females and HR = 1.52, CI: 1.39; 1.66 in males). Analyses using non-discretized DC and gingivitis variables overall supported these findings, though DC was not statistically significant in males after adjustment for education (Supplementary Table 2). Only one interaction was significant (Supplementary Table 3); in females diagnosed with T2D before age 40 having moderate DCS with a moderate to high GS is associated with a lower interaction HR for T2D (HR = 0.79, CI: 0.63; 0.98).

**Table 2:** Main effects for the association between T2D and the highest registered DCS and GS at any point in time in SCOR

	Males		Females	
	HR (CI)	P	HR (CI)	P
No age restriction				
DCS: Low	1.00 (ref)	**-**	1.00 (ref)	**-**
Moderate	1.08 (1.02; 1.14)	0.01	1.04 (0.97; 1.12)	0.31
High	1.09 (1.02; 1.17)	0.01	1.07 (0.98; 1.17)	0.11
Severe	1.15 (1.04; 1.27)	0.005	1.19 (1.06; 1.35)	0.003
GS: Low	1.00 (ref)	**-**	1.00 (ref)	-
Moderate	1.23 (1.18; 1.28)	< 0.001	1.24 (1.18; 1.29)	< 0.001
High	1.59 (1.47; 1.72)	< 0.001	1.87 (1.68; 2.08)	< 0.001
Age < 40				
DCS: Low	1.00 (ref)	**-**	1.00 (ref)	-
Moderate	1.02 (0.91; 1.14)	0.77	0.94 (0.84; 1.05)	0.25
High	0.94 (0.82; 1.08)	0.36	0.88 (0.77; 1.01)	0.07
Severe	0.93 (0.75; 1.15)	0.48	0.98 (0.79; 1.19)	0.81
GS: Low	-	-	-	-
Moderate	1.28 (1.18; 1.39)	< 0.001	1.17 (1.08; 1.27)	< 0.001
High	1.84 (1.58; 2.15)	< 0.001	1.94 (1.63; 2.30)	< 0.001
Age ≥ 40				
DCS: Low	1.00 (ref)	**-**	1.00 (ref)	-
Moderate	1.09 (1.03; 1.17)	0.007	1.13 (1.02; 1.25)	0.01
High	1.14 (1.06; 1.24)	< 0.001	1.23 (1.09; 1.38)	0.005
Severe	1.23 (1.09; 1.38)	< 0.001	1.37 (1.17; 1.59)	< 0.001
GS: Low	1.00 (ref)	-	1.00 (ref)	-
Moderate	1.21 (1.15; 1.27)	< 0.001	1.28 (1.21; 1.36)	< 0.001
High	1.52 (1.39; 1.66)	< 0.001	1.83 (1.59; 2.10)	< 0.001

Highest achieved level of education (observed between 25–30 years) used as strata

*CI* confidence interval, *DCS* dental caries score, *GS* gingivitis score, *HR* hazard ratio, *P* p-value, *ref* reference group

Testing at a 95% confidence level

### The association of T2D with the age at the highest registered levels of oral disease, duration, and severity over time of dental caries and gingivitis

In females, neither age at disease, severity over time, nor duration of DC were related to the incidence of T2D (Table 3, unadjusted estimates in Supplementary Table 4). In males, having a moderate severity DCS for the duration of the time in SCOR was associated with a higher incidence of T2D (HR = 1.32, CI: 1.07; 1.64), while timing and duration of DC were not. Having the highest GS registered at or above age 12 was associated with a higher incidence of T2D (HR = 1.37, CI: 1.13; 1.65) in females, and in males (HR = 1.25, CI: 1.08; 1.45). In males high severity GS throughout the time in SCOR was associated with an HR of 1.61 (CI: 1.19; 2.16) compared to a low GS, and having gingivitis throughout the time in SCOR, e.g. within all three defined age-ranges and regardless of the severity, was associated with an HR of 1.24 (CI: 1.06; 1.45) compared to having gingivitis at two or less periods in time. Similar patterns appeared in the effect of gingivitis on the HRs of T2D for females, though these did not reach statistical significance.

**Table 3:** Secondary (subset) analyses of the hazard of T2D by age at highest registered DCS and GS, duration, and severity over time of DC and gingivitis

	Males		Females	
	HR (CI)	P	HR (CI)	P
DC				
Age at highest registered DCS				
Below age 12	1.00 (ref)	-	1.00 (ref)	-
Age 12 or above	1.01 (0.84; 1.22)	0.91	1.10 (0.89; 1.35)	0.37
DCS over time				
Low	1.00 (ref)	–	1.00 (ref)	–
Moderate	1.32 (1.07; 1.64)	0.01	0.91 (0.72; 1.14)	0.39
High—Severe	1.28 (0.95; 1.71)	0.10	0.89 (0.64; 1.24)	0.49
DC duration				
Up to 2 periods in time	1.00 (ref)	–	1.00 (ref)	–
3 periods in time	1.07 (0.89; 1.29)	0.47	1.09 (0.89; 1.35)	0.40
Gingivitis				
Age at highest registered GS				
Below age 12	1.00 (ref)	–	1.00 (ref)	–
Age 12 or above	1.25 (1.08; 1.45)	0.003	1.37 (1.13; 1.65)	< 0.001
GS over time				
Low	1.00 (ref)	–	1.00 (ref)	–
Moderate	1.19 (1.004; 1.42)	0.04	1.11 (0.94; 1.32)	0.23
High	1.61 (1.19; 2.16)	0.002	1.41 (0.93; 2.15)	0.10
Gingivitis duration				
Up to 2 periods in time	1.00 (ref)	–	1.00 (ref)	–
3 periods in time	1.24 (1.06; 1.45)	0.008	1.06 (0.89; 1.26)	0.47

Highest achieved level of education (observed between 25 and 30 years) used as strata. Testing at a 95% confidence level

*CI* confidence interval, *DCS* dental caries score, *GS* gingivitis score, *HR* hazard ratio, *P* p-value

## Discussion

In this registry-based study we found a higher incidence of T2D among adult Danes that had DC and gingivitis in childhood, thereby confirming our main hypothesis for this study. In both sexes, having one’s highest registered level of gingivitis after the age of 12 was associated with a higher incidence of T2D in adulthood. The results should be interpreted with caution due to residual confounding.

The highest HRs of T2D were found with high levels of GS. This could be explained by the low-grade inflammation inferred by gingivitis, as it may cause a long-lasting reprogramming of granulopoiesis resulting in a persisting neutrophil hyper-responsiveness, in a similar way as has been suggested for periodontitis [24]. In adipose tissues, neutrophils are then activated and release a range of inflammatory factors, contributing to the development of insulin resistance. In line with this theory of inflammation, one may speculate if the greater effect of gingivitis in teenagers compared to children in this study, is due to a stronger immune response to low-grade inflammation of the more mature teenage immune-system [25].

While research on the genetics of gingivitis and T2D is lacking, there is evidence that periodontitis and T2D are genetically linked [26, 27]. Given the similarities between the two oral diseases [5], these genes could also play a role in the association to T2D observed in this study. Evidence suggests that DC has a high heritability and that DC correlates with several traits of metabolic disease on a genetic level [28, 29]. Thus, the associations of DC and T2D in our study may have a considerable genetic component.

In Denmark, there has historically been a great focus on reducing the DC incidence and prevalence in children and adolescents. Meanwhile, gingivitis has received little attention. Today registration of gingivitis in SCOR has long ceased to be a mandatory [17]. Our study suggests that the impact of gingivitis may be as relevant as the impact of DC for the subsequent incidence of T2D. One interpretation of this could be that efficient prevention of gingivitis in childhood, including targeted education and oral hygiene instructions in high-risk individuals, may have a long-term protective effect on T2D development later in life.

A significant limitation of our study is that the registry data lack information about potential confounders. We do not have direct measurements of lifestyle factors e.g. smoking and eating habits. Infrequent tooth-brushing in the teenage years has been shown to correlate with higher rates of smoking, an obesogenic lifestyle, and a lower level of education later in life [30]. Accordingly, using the highest achieved level of education as strata considerably lowered the HRs in this study. While hopefully having captured some of the confounding effects of lifestyle-factors, our estimates are bound to be affected by residual confounding. Thus, we cannot ultimately conclude that DCS and GS are causes of T2D. Studies with individual level information on lifestyle factors are warranted.

We were not able to account for individuals travelling in and out of Denmark. We may assume that individuals who leave the country, develop diabetes, and then return, will still be registered as having T2D, as anyone prescribed medication via the Danish healthcare system will be registered in RUKS. The prevalence of T2D in our study population corresponds well with the nationwide disease prevalence in the period [31]. Thus, most T2D-cases have likely been registered, though there may be a delay in the date of diagnosis for some individuals, somewhat artificially increasing survival times.

The subset of the study population used for the analyses presented in Table 3 excludes the oldest individuals in our dataset. Due to being born between 1963 and 1966 they do not have registrations in SCOR between ages 0–5, as SCOR didn’t exist at the time. As an effect of their current age, the excluded individuals are the ones at the highest risk of T2D in the available time-at-risk. This may increase the probability of finding no associations between oral health and T2D in the subset analyses and produce more uncertain effect estimates.

Strengths of our study include the large sample size, with nationwide registrations of both exposures and outcome, the length of follow-up, and the detailed registrations of oral health.

In conclusion, we report evidence for the association between childhood experience of DC and gingivitis with receiving a T2D diagnosis in adulthood. Our findings suggest the need for a greater awareness of gingivitis in childhood dental care. However, studies adjusting for confounding variables to a greater extent than possible here are needed.

## Supplementary Information

Below is the link to the electronic supplementary material.

Supplementary file1 (DOCX 411 KB)

## Data Availability

The full data from SCOR underlying the present article are available from The Danish Health Data Authority [32], but restrictions apply to the availability of these data. The R-script created for cleaning data and generating the analyses presented in the present paper can be found on GitHub [33].
