# Supplementary material for: A nationwide registry-based cohort study of the association between childhood dental caries and gingivitis with type 2 diabetes in adulthood
Source: Acta Diabetol. 2025 Jan 13;62(8):1195–204. doi: 10.1007/s00592-024-02437-4 (PMC12364746; doi:10.1007/s00592-024-02437-4)
Supplement: Supplementary file 1 — Supplementary file1 (DOCX 411 KB) [file 592_2024_2437_MOESM1_ESM.docx]

**Supplementary materials**

Supplementary Figure 1. Distribution of DCS and GS in the study population and amongst those with T2D, stratified by sex
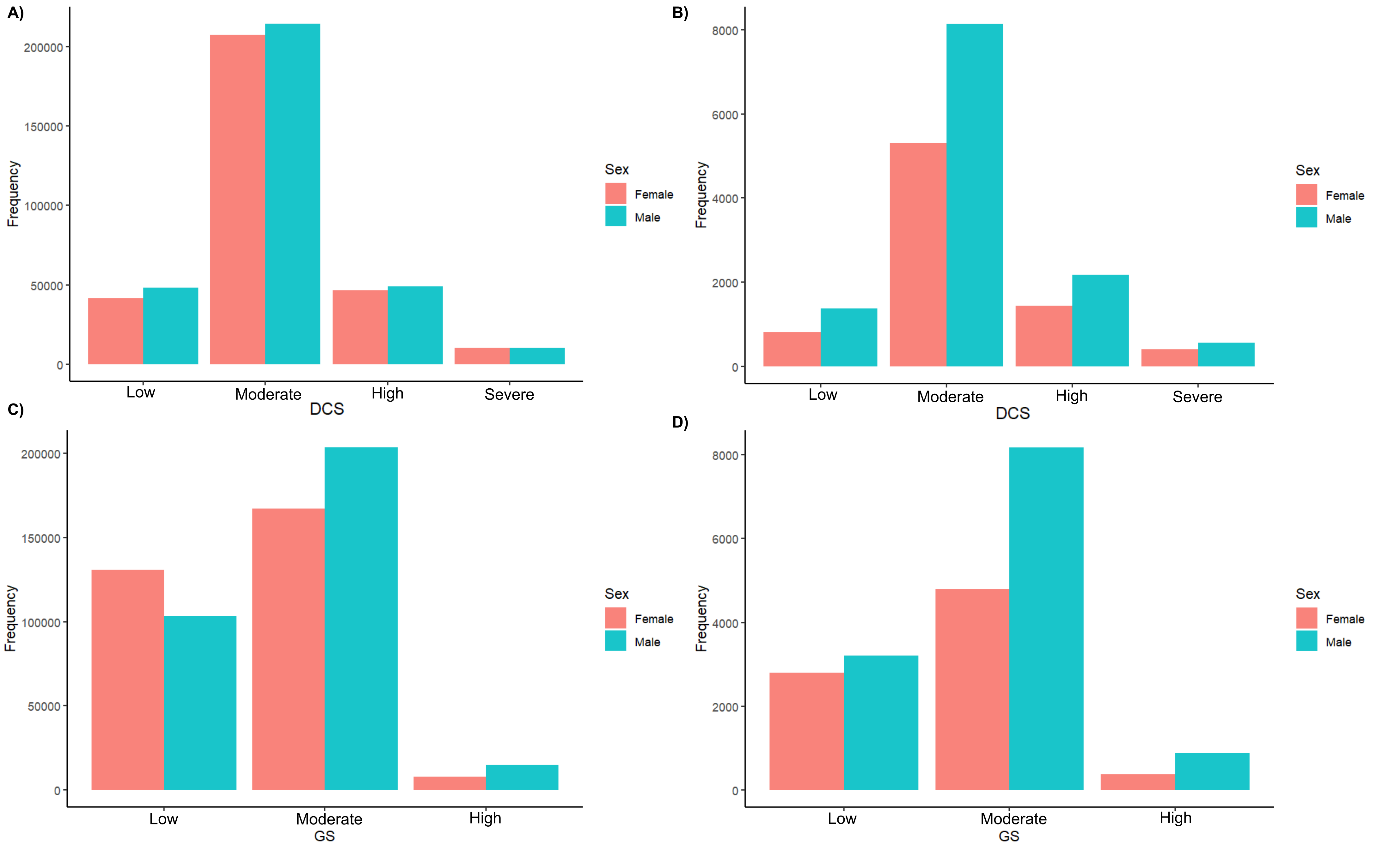


Supplementary Figure 1 legend. Panel A) DCS distribution in the study population. Panel B) DCS distribution in those with T2D. Panel C) GS distribution in the study population. Panel D) GS distribution in those with T2D. Abbreviations: DCS = Dental caries score, GS = Gingivitis score, T2D = Type 2 diabetes.

Supplementary Figure 2. The distribution of GS by DCS in the study population and amongst those with T2D, stratified by sex
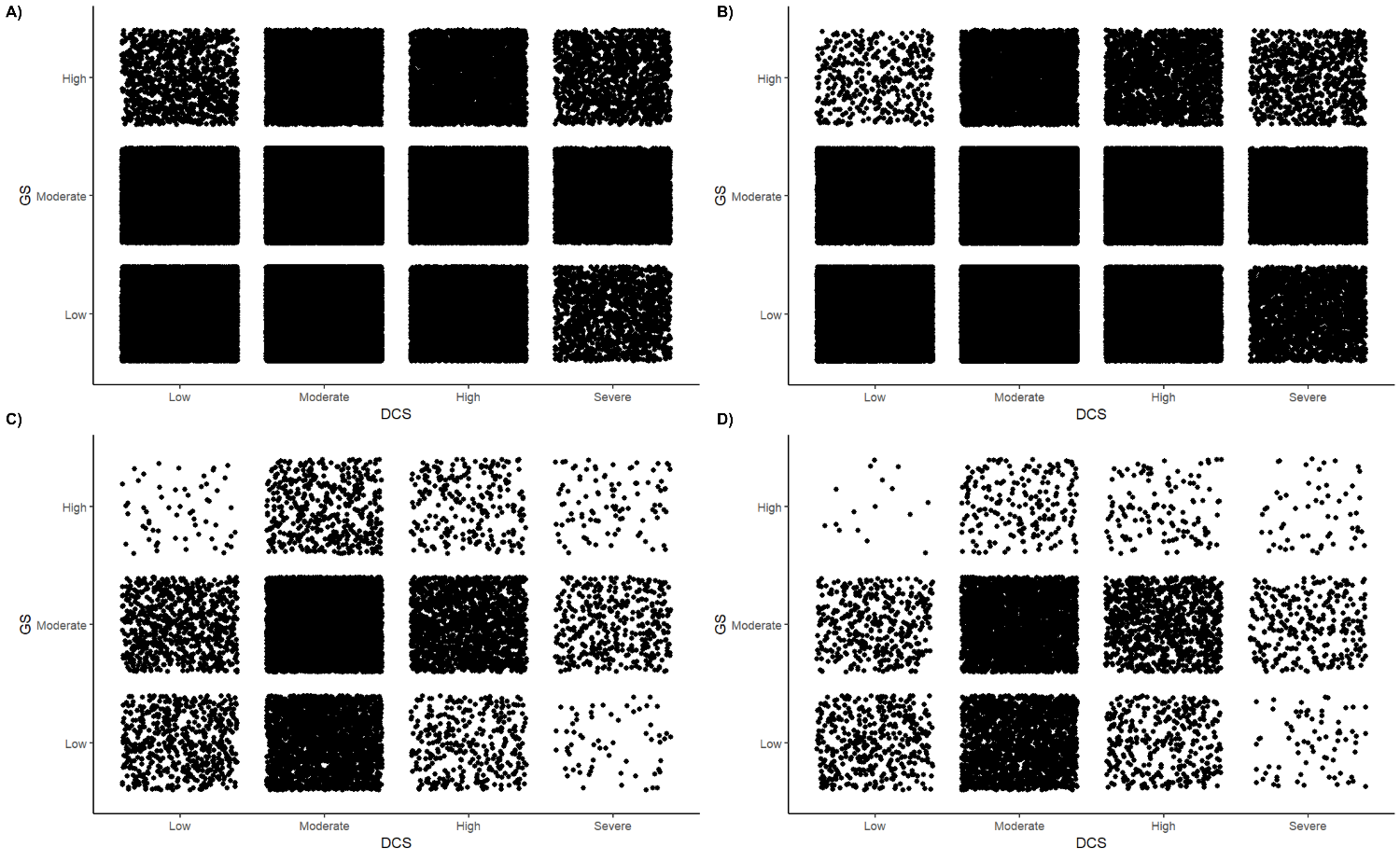


Supplementary Figure 2 legend. Panel A) Distribution of GS by DCS in the male study population. Panel B) Distribution of GS by DCS in the female study population. Panel C) Distribution of GS by DCS in males with T2D. Panel D) Distribution of GS by DCS in females with T2D. Abbreviations: DCS = Dental caries score, GS = Gingivitis score, T2D = Type 2 diabetes.

Supplementary Table 1. Unadjusted estimates for the association between T2D and childhood caries and gingivitis

|  | **Males** | | **Females** | |
| --- | --- | --- | --- | --- |
|  | **HR (CI)** | **P** | **HR (CI)** | **P** |
| **No age restriction** |  |  |  |  |
| DCS: Low | 1.00 (ref) | **-** | 1.00 (ref) | **-** |
| *Moderate* | 1.18 (1.12; 1.13) | < 0.001 | 1.16 (1.07; 1.24) | < 0.001 |
| *High* | 1.27 (1.19; 1.36) | < 0.001 | 1.28 (1.17; 1.39) | < 0.001 |
| *Severe* | 1.42 (1.29; 1.57) | < 0.001 | 1.52 (1.35; 1.72) | < 0.001 |
| GS: Low | 1.00 (ref) | **-** | 1.00 (ref) | - |
| *Moderate* | 1.30 (1.18; 1.35) | < 0.001 | 1.32 (1.26; 1.39) | < 0.001 |
| *High* | 1.82 (1.69; 1.97) | < 0.001 | 2.22 (1.99; 2.47) | < 0.001 |
| **Age < 40** |  |  |  |  |
| DCS: Low | 1.00 (ref) | **-** | 1.00 (ref) | - |
| *Moderate* | 1.12 (1.00; 1.26) | 0.04 | 1.03 (0.93; 1.15) | 0.58 |
| *High* | 1.10 (0.96; 1.27) | 0.17 | 1.04 (0.90; 1.19) | 0.62 |
| *Severe* | 1.16 (0.93; 1.44) | 0.19 | 1.22 (0.99; 1.49) | 0.05 |
| GS: Low | - | - | - | - |
| *Moderate* | 1.37 (1.25; 1.49) | < 0.001 | 1.25 (1.16; 1.35) | < 0.001 |
| *High* | 2.15 (1.85; 2.51) | < 0.001 | 2.29 (1.93; 2.72) | < 0.001 |
| **Age** ≥ **40** |  |  |  |  |
| DCS: Low | 1.00 (ref) | **-** | 1.00 (ref) | - |
| *Moderate* | 1.20 (1.13; 1.29) | < 0.001 | 1.27 (1.14; 1.40) | < 0.001 |
| *High* | 1.33 (1.23; 1.44) | < 0.001 | 1.48 (1.32; 1.66) | < 0.001 |
| *Severe* | 1.51 (1.35; 1.69) | < 0.001 | 1.76 (1.51; 2.05) | < 0.001 |
| GS: Low | 1.00 (ref) | - | 1.00 (ref) | - |
| *Moderate* | 1.28 (1.22; 1.34) | < 0.001 | 1.37 (1.29; 1.46) | < 0.001 |
| *High* | 1.73 (1.59; 1.89) | < 0.001 | 2.18 (1.90; 2.50) | < 0.001 |

Supplementary Table 1 legend. Abbreviations: CI = 95% confidence interval, DCS = Dental caries score, GS = Gingivitis score, HR = Hazard ratios.

Supplementary Table 2. The association between T2D and childhood caries and gingivitis used as continuous variables

|  | **Males** | | **Females** | |
| --- | --- | --- | --- | --- |
|  | **HR (CI)** | **P** | **HR (CI)** | **P** |
| **Unadjusted** |  |  |  |  |
| dmft/DMFT | 1.01 (1.01; 1.02) | < 0.001 | 1.02 (1.01; 1.03) | < 0.001 |
| Gingivitis index score | 1.09 (1.08; 1.09) | < 0.001 | 1.10 (1.10; 1.11) | < 0.001 |
| **Adjusted*** |  |  |  |  |
| dmft/DMFT | 1.00 (0.99; 1.00) | 0.47 | 1.01 (1.00; 1.01) | 0.04 |
| Gingivitis index score | 1.07 (1.06; 1.08) | < 0.001 | 1.08 (1.07; 1.09) | < 0.001 |

Supplementary Table 2 legend. *Highest achieved level of education between ages 25 and 30 used as strata. Abbreviations: CI = 95% confidence interval, dmft/DMFT = highest registered number decayed, missing or filled teeth in either the temporary or permanent dentition, HR = Hazard ratios.

Supplementary Table 3. Interaction analyses of the association between T2D and the highest registered DCS and GS at any point in time in SCOR.

|  | **Males** | | **Females** | |
| --- | --- | --- | --- | --- |
|  | **HR (CI)** | **P** | **HR (CI)** | **P** |
| **No age-restriction** |  |  |  |  |
| DCS: Low | 1.00 (ref) | **-** | 1.00 (ref) | **-** |
| *Moderate* | 1.13 (0.88; 1.02) | 0.01 | 1.09 (0.98; 1.21) | 0.11 |
| *High/Severe* | 1.11 (0.90; 1.26) | 0.12 | 1.18 (1.03; 1.36) | 0.02 |
| GS: Low | 1.00 (ref) | **-** | 1.00 (ref) | **-** |
| *Moderate/High* | 1.32 (1.18; 1.47) | < 0.001 | 1.39 (1.21; 1.59) | < 0.001 |
| DCS×GS: | - | - | - | - |
| *Moderate×Moderate/High* | 0.99 (0.83; 1.06) | 0.25 | 0.91 (0.78; 1.05) | 0.19 |
| *High/Severe×Moderate/High* | 0.97 (0.83; 1.14) | 0.93 | 0.89 (0.75; 1.06) | 0.21 |
| **Age < 40** |  |  |  |  |
| DCS: low | 1.00 (ref) | **-** | 1.00 (ref) | **-** |
| *Moderate* | 1.16 (0.96; 1.41) | 0.14 | 1.07 (0.91; 1.25) | 0.04 |
| *High/Severe* | 0.99 (0.75; 1.33) | 0.99 | 0.99 (0.79; 1.24) | 0.92 |
| GS: low | 1.00 (ref) | **-** | 1.00 (ref) | **-** |
| *Moderate/Severe* | 1.53 (1.24; 1.89) | < 0.001 | 1.47 (1.21; 1.79) | < 0.001 |
| DCS×GS: | - | - | - | - |
| *Moderate×Moderate/High* | 0.83 (0.65; 1.05) | 0.11 | 0.79 (0.63; 0.98) | 0.03 |
| *High/Severe×Moderate/High* | 0.91 (1.66; 1.26) | 0.58 | 0.84 (0.64; 1.11) | 0.22 |
| **Age** ≥ **40** |  |  |  |  |
| DCS: low | 1.00 (ref) | **-** | 1.00 (ref) | **-** |
| *Moderate* | 1.12 (1.00; 1.24) | 0.04 | 1.12 (0.97; 1.29) | 0.12 |
| *High/Severe* | 1.14 (0.98; 1.31) | 0.08 | 1.32 (1.11; 1.57) | 0.001 |
| GS: low | 1.00 (ref) | **-** | 1.00 (ref) | **-** |
| *Moderate/Severe* | 1.25 (1.09; 1.41) | < 0.001 | 1.30 (1.08; 1.58) | 0.006 |
| DCS×GS: | - | - | - |  |
| *Moderate×Moderate/High* | 0.98 (0.85; 1.12) | 0.75 | 1.02 (0.83; 1.25) | 0.87 |
| *High/Severe×Moderate/High* | 1.04 (0.87; 1.23) | 0.69 | 0.95 (0.76; 1.19) | 0.68 |

Supplementary Table 3 legend. Using the highest achieved level of education (observed between 25-30 years) as strata. Testing at a 95% confidence level. Abbreviations: CI = confidence interval, DCS = dental caries score, GS = gingivitis score, HR = hazard ratio, P = p-value, ref = reference group.

Supplementary Table 4. Unadjusted estimates for the association of T2D with the age at the highest registered levels of oral disease, duration, and severity over time of dental caries and gingivitis

|  | **Males** | | **Females** | |
| --- | --- | --- | --- | --- |
|  | **HR (CI)** | **P** | **HR (CI)** | **P** |
| **DC** | | | | |
| Age at highest registered DCS | | | | |
| *Below age 12* | 1.00 (ref) | - | 1.00 (ref) | - |
| *Age 12 or above* | 1.04 (0.87; 1.25) | 0.65 | 1.12 (0.91; 1.37) | 0.29 |
| DCS over time | | | | |
| *Low* | 1.00 (ref) | - | 1.00 (ref) | - |
| *Moderate* | 1.44 (1.16; 1.78) | < 0.001 | 1.07 (0.77; 1.49) | 0.97 |
| *High - Severe* | 1.49 (1.12; 2.05) | < 0.01 | 1.07 (0.93; 1.42) | 0.71 |
| DC duration | | | | |
| *Up to 2 periods in time* | 1.00 (ref) | - | 1.00 (ref) | - |
| *3 periods in time* | 1.13 (0.94; 1.37) | 0.20 | 1.15 (0.93; 1.42) | 0.21 |
| **Gingivitis** | | | | |
| Age at highest registered GS | | | | |
| *Below age 12* | 1.00 (ref) | - | 1.00 (ref) | - |
| *Age 12 or above* | 1.27 (1.09; 1.47) | <0.01 | 1.41 (1.17; 1.69) | < 0.001 |
| GS over time | | | | |
| *Low* | 1.00 (ref) | - | 1.00 (ref) | **-** |
| *Moderate* | 1.29 (1.09; 1.54) | <0.01 | 1.20 (1.01; 1.43) | 0.03 |
| *High* | 1.94 (1.44; 2.60) | <0.001 | 1.71 (1.12; 2.59) | 0.01 |
| Gingivitis duration | | | | |
| *Up to 2 periods in time* | 1.00 (ref) | - | 1.00 (ref) | - |
| *3 periods in time* | 1.29 (1.09; 1.51) | <0.01 | 1.11 (0.93; 1.31) | 0.24 |

Supplementary Table 4 legend. Testing at a 95% confidence level. Abbreviations: CI = confidence interval, DCS = dental caries score, GS = gingivitis score, HR = hazard ratio, P = p-value.
